# Germline recombination by conditional gene targeting with Parvalbumin-Cre lines

**DOI:** 10.3389/fncir.2013.00168

**Published:** 2013-10-16

**Authors:** Yohei Kobayashi, Takao K. Hensch

**Affiliations:** ^1^Department of Molecular and Cellular Biology, Center for Brain Science, Harvard UniversityCambridge, MA, USA; ^2^Department of Neurology, F. M. Kirby Neurobiology Center, Harvard Medical School, Boston Children's HospitalMA, USA

**Keywords:** Cre, conditional targeting, knock-out mouse, germline, testis, parvalbumin

Conditional gene targeting allows us to study gene function in specific tissues or cell types. This is commonly achieved by Cre DNA recombinase and its 34–base pair target sequences called loxP sites. Through the efforts of individual labs and large-scale projects, a sizable collection of Cre mouse lines has been generated to express or delete specific genes in a wide range of cell types throughout the nervous system (Madisen et al., 2010; Taniguchi et al., 2011). Typically, the specificity of Cre transgene expression is controlled by tissue or cell-type promoters. However, increasing evidence has revealed that the desired Cre expression pattern is not always guaranteed (Schmidt-Supprian and Rajewsky, 2007).

Here, we show an example of the undesired recombination in a Cre line whose expression is controlled by the parvalbumin (*Pvalb*) promoter. Currently, two *Pvalb-Cre* lines are available: the *Pvalb-IRES-Cre* (Hippenmeyer et al., 2005) and *Pvalb-2A-Cre* (Madisen et al., 2010) in which either *IRES-Cre* or *2A-Cre* sequence is inserted by knock-in into the *Pvalb* 3′ untraslated region. Both Cre lines have been widely used to induce or delete genes of interest in parvalbumin (PV)-positive GABAergic neurons in the brain. However, PV is expressed not only in brain but also in other tissues, including testis in particular (Kagi et al., 1987), suggesting that undesired germline recombination could occur in the *Pvalb-Cre* testis.

To examine recombination, we crossed *Ai32* (*ChR2* (H134R)*-EYFP*) line whose EYFP expression can be detected after recombination (Madisen et al., 2012) with either the *Pvalb-IRES-Cre* or *Pvalb-2A-Cre* line. In the *Pvalb-2A-Cre^+/−^* background, strong EYFP expression was observed in Leydig cells (Figures 1A,C, arrows) and spermatids in the seminiferous tubules (Figures 1A,C, asterisks) in which PV was expressed (Figure 1B). In contrast, in the *Pvalb-IRES-Cre^+/−^* background, although EYFP was slightly expressed in Leydig cells, EYFP was absent from spermatids (Figures 1D,E). This probably reflects weaker expression of Cre under control of the *IRES* as compared to the *2A* sequence (Madisen et al., 2010).

**Figure 1:**
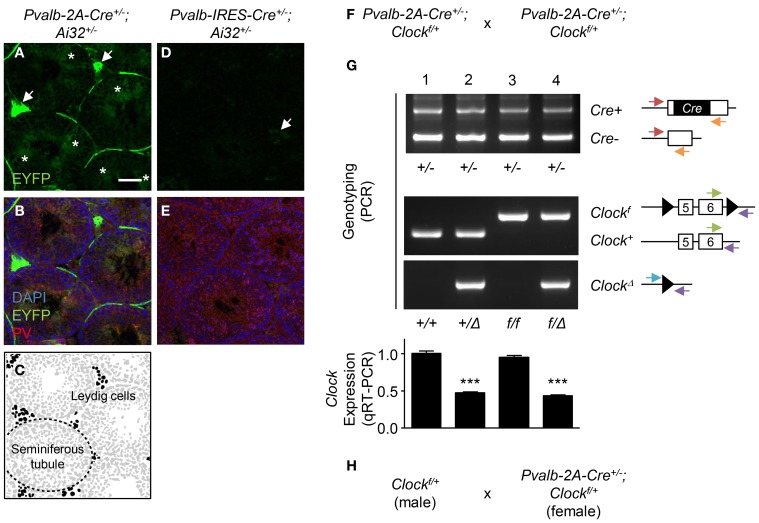
**Germline recombination in *Pvalb-2A-Cre testis*. (A–E)** Expression of EYFP in *Pvalb-2A-Cre^+/−^; Ai32^+/−^* **(A–C)** or *Pvalb-IRES-Cre^+/−^; Ai32^+/−^* testes **(D,E)**. Testes were co-stained with DAPI and anti-parvalbumin (PV) antibody **(B,E)**. One of the seminiferous tubules (dashed line) and Leydig cells (black) are depicted **(C)**. Arrows, EYFP-expressing Leydig cells. Asterisks, EYFP-expressing spermatids. Scale bar, 100 μm. **(F)** Breeding strategy to generate PV-cell specific *Clock* conditional knock-out mice using *Pvalb-2A-Cre* line. **(G)** Genotyping and qRT-PCR analysis of the progeny obtained by **(F)**. Genotyping was performed to detect *Cre* positive (*Cre*+) or negative (*Cre^−^*), floxed *Clock* (*Clock^f^*) or wild-type (*Clock^+^*), or deletion of *Clock* (*Clock*^Δ^). Arrows indicate location and direction of primers for PCR genotyping. Expression of *Clock* in neocortex was analyzed by qRT-PCR (graph, 8–12 mice each). ^***^P < 0.001 (One-Way ANOVA, Dunnett's *post-hoc* analysis). Values are mean ± s.e.m. **(H)** Modified breeding strategy to avoid germline recombination when generating PV-cell specific *Clock* conditional knock-out mice.

In the conditional knock-out strategy, such recombination in testis should be avoided because it could induce a “total” knock-out of the gene. One example of this undesired recombination by the *Pvalb-2A-Cre* line is shown in Figures 1F,G. To knock-out the *Clock* gene, *Pvalb-2A-Cre^+/−^; Clock^f/+^* mice were crossed with each other (Figure 1F), and genotypes of its progeny were determined for the *Cre* and *Clock* alleles (Figure 1G). In some offspring, undesired deletion of the *Clock* gene (*Clock*^Δ^) was detected (Figure 1G, lane 2 and 4).

In our experience, such undesired recombination happened in 50% of the progeny using this strategy (24 out of 48 mice). Quantitative RT-PCR (qRT-PCR) analysis of brain samples further confirmed deletion of the *Clock* gene with expression decreased by half in the recombined offspring (Figure 1G, graph, lane 2 and 4), while only ~5% decrease of *Clock* expression was seen in the expected conditional progeny (Figure 1G, graph, lane 3). To minimize this undesired recombination, breeding Cre-negative males (*Clock^f/+^*) with Cre-positive females (*Pvalb-2A-Cre^+/−^; Clock^f/+^*) was performed (Figure 1H). In this strategy, the undesired recombination frequency became less than 5%, although it was not completely abolished for unknown reasons.

Such germline recombination is difficult to track in the literature, but it has been noted across several Cre lines widely used in the field of neuroscience, including *Camk2a-Cre* (Friedel et al., 2011), *Nestin-Cre* (Friedel et al., 2011; Harno et al., 2013), *Emx1-Cre* (Zeller et al., 2008), and *Pcp2-Cre* (Tsai et al., 2012). Given this situation, these could simply be the tip of an iceberg. In order to avoid undesired outcomes, here we propose several precautionary measures:

First, expression of Cre outside the region of interest, especially in the germline, should be investigated. For several Cre lines, expression patterns are available in common databases such as The Jackson Laboratory Cre Repository (http://cre.jax.org/data.html) or Cre Portal (http://www.creportal.org/). It should be noted that the recombination pattern has to be evaluated for each floxed line, because recombination efficiency depends upon the region on the chromosome. Second, a breeding strategy should be set up in a way that minimizes the occurrence of germline recombination. Third, deletion of the floxed genes must be analyzed by genotyping as shown in Figure 1G, which is often omitted in the standard conditional knock-out strategy. This is particularly important to avoid misinterpretation of phenotypes due to unexpected recombination in other cells than the desired target.

## Materials and methods

### Mice

C57BL/6J (JAX no. 000664), *Pvalb-2A-Cre* (JAX no. 012358) (Madisen et al., 2010), *Pvalb-IRES-Cre* (JAX no. 008069) (Hippenmeyer et al., 2005), *Ai32* (JAX no. 012569) (Madisen et al., 2012), and *Clock^flox^* (JAX no. 010490) (Debruyne et al., 2006) mice were purchased from Jackson Laboratory. All mice were on a C57BL/6J background. For examining recombination in testis, *Pvalb-2A-Cre^+/+^* or *Pvalb-IRES-Cre^+/+^* mice were crossed with *Ai32^+/−^* mice to generate *Pvalb-2A-Cre^+/−^; Ai32^+/−^* or *Pvalb-IRES-Cre^+/−^; Ai32^+/−^* mice. For examining deletion and expression level of *Clock* gene, *Pvalb-2A-Cre^+/+^* mice were crossed with *Clock^f/f^* mice to generate *Pvalb-2A-Cre^+/−^; Clock^f/+^* mice, and these progeny were then crossed with each other. Animal housing and experimental procedures were approved (AEP28-19) and followed guidelines of the Harvard University Institutional Animal Care and Use Committee.

## Immunohistochemistry

Testes were decapsulated and embedded in OCT compound (Sakura Finetek USA Inc), quickly frozen and sectioned on a Cryostat (Leica). Cryo- sections (7 μm) were fixed in 4% (wt/vol) paraformaldehyde in 0.1 M phosfate buffer for 15 min at room temperature, washed with PBS thrice, and blocked with buffer (10% normal goat serum and 0.1% Triton X-100 (vol/vol) in PBS) for 1 h at room temperature. Incubation with rabbit anti-parvalbumin (Swant, 1:1000) was performed in blocking buffer overnight at 4°C, followed by incubation with Alexa Fluor 488 Goat Anti-Rabbit IgG (Molecular Probes, 1:400) in blocking buffer overnight at 4°C. Stained sections were mounted with 4′,6-diamidino-2-phenylindole (DAPI) and visualized by confocal microscopy (FV1000, Olympus).

## PCR genotyping

Genomic DNA was extracted from ear punches and PCR genotyping performed using REDExtract-N-Amp Tissue PCR Kit (Sigma). Primers used in this study were described elsewhere (Debruyne et al., 2006).

## Real-time quantitative RT-PCR

Total RNA was isolated from the neocortex with *mir*Vana miRNA Isolation Kit (Ambion), and any contaminating DNA removed by TURBO DNA-*free* Kit (Invitrogen) according to manufacturer's instructions. First-strand cDNA was synthesized from total RNA using High Capacity RNA-to-cDNA Kit (Invitrogen) according to manufacturer's instructions. Real-time quantitative PCR was performed using TaqMan Gene Expression Assay (Applied Biosystems) on a StepOnePlus Real-Time PCR System (Applied Biosystems). TaqMan probes used in this study were for *Clock* exon 5–6 (Mm00455940_g1) and *Gapdh* (4352932E). Relative expression of target genes was determined by the 2^−ΔΔ*Ct*^ method.
